# Single-dose Tadalafil Reduces Opening Urethral Pressure: A Randomized, Double-blind, Placebo-controlled, Crossover Trial in Healthy Women

**DOI:** 10.1007/s00192-024-05757-9

**Published:** 2024-03-08

**Authors:** Thea Christoffersen, Troels Riis, David P. Sonne, Niels Klarskov

**Affiliations:** 1 Department of Clinical Pharmacology, Copenhagen University Hospital - Bispebjerg and Frederiksberg, Bispebjerg Bakke 23, 2400 Copenhagen, Denmark; 2 Department of Clinical Medicine, University of Copenhagen, Copenhagen, Denmark; 3 Department of Gynecology and Obstetrics, Copenhagen University Hospital - Herlev and Gentofte, Herlev, Denmark

**Keywords:** Female, Impaired voiding, Phosphodiesterase inhibitors, Tadalafil, Urethral pressure reflectometry, Urinary retention

## Abstract

**Introduction and Hypothesis:**

Phosphodiesterase enzymes are widely distributed in female urogenital tissues. Yet, the understanding of their physiological roles and the impact of phosphodiesterase inhibitors on lower urinary tract symptoms in women remains limited. Current hypotheses are conflicting: one suggests that vasodilation might expand the periurethral vascular plexus, leading to increased urethral pressure, whereas the other proposes a relaxation of urethral musculature, resulting in decreased pressure. To further clarify this, we investigated the effect of tadalafil on the opening urethral pressure and voiding function in healthy women.

**Methods:**

We conducted a randomized, double-blind, placebo-controlled crossover trial involving 24 healthy women. Participants were randomly assigned to receive a single dose of tadalafil (40 mg) or placebo during their initial visit and then switched to the alternative treatment during their second visit. Opening urethral pressure was measured with urethral pressure reflectometry during both resting and squeezing conditions of the pelvic floor. Subsequently, voiding parameters were recorded.

**Results:**

Compared with placebo, a single dose of tadalafil significantly reduced opening urethral pressure during both resting (-6.8 cmH_2_0; 95% confidence interval [CI], -11.8 to -1.9; *p* = 0.009) and squeezing conditions (-8.8 cmH_2_0; 95% CI, -14.6 to -3.1; *p* = 0.005). Voiding parameters did not show significant differences (average flow rate: -0.8 ml/s [95% CI, -2.0 to 0.4; *p* = 0.2]; maximum flow rate: -1.7 ml/s [95% CI, -4.8 to 1.5; *p* = 0.3]).

**Conclusions:**

A single dose of 40 mg tadalafil moderately reduced urethral pressure in healthy women, without affecting voiding parameters. The clinical implications of this are yet to be determined.

## Introduction

The serendipitous discovery of the effect of the selective phosphodiesterase type 5 (PDE5) inhibitor sildenafil on male erectile dysfunction in the late 1990s led to extensive research on the physiological actions of the PDE isoenzymes and the potential therapeutic utility of various pharmacological PDE inhibitors [1]. The main findings that have led to additional therapeutic indications include the subjective improvements of lower urinary tract (LUT) symptoms due to benign prostatic hyperplasia and increased walking distance in patients with pulmonary arterial hypertension [2, 3].

Phosphodiesterases constitute a superfamily of hydrolytic enzymes that degrade the second messengers cAMP and cGMP. Intracellularly, cAMP and cGMP trigger signaling pathways leading to a range of processes, including smooth muscle relaxation. Nitric oxide (NO) is pivotal in these pathways, mediating cGMP synthesis. PDE inhibitors enhance the NO-dependent smooth muscle relaxation by inhibiting PDEs [4].

To date, the most pronounced effects of PDE inhibition have been demonstrated in the male urogenital tract and consequently, most research activities have focused on new therapeutic applications for male LUT dysfunctions. Despite the knowledge of widespread PDE enzyme distribution in female urogenital tissues [5], little is known about the physiological effects on the female urogenital system and the potential effects of PDE inhibitors in women.

Preclinical studies suggest an important role of the NO/cGMP pathways in the regulation of the female urethral sphincter tonus [6, 7]; however, the outcomes are inconclusive. Although in vitro experiments on isolated female urethral muscle strips have suggested a smooth muscle relaxant effect of PDE5 inhibitors [5], an experimental study in anesthetized female rats found that intra-urethral administration of the PDE (type 1, 5, 6, and 9) inhibitor zaprinast increased the tone of the striated urethral sphincter and thereby the baseline urethral pressure [8]. Further, sildenafil did not improve sphincter relaxation compared with placebo in women with urinary retention due to abnormal urethral sphincter activity (Fowler’s syndrome) [9].

Considering the dense vascular plexus surrounding the female urethra, which contributes to the maintenance of the urethral pressure during the storage phase [10], selective PDE5 inhibitors may potentially potentiate the relaxation of vascular smooth muscle cells thereby increasing the volume of the vascular plexus and, as a result, increase the urethral pressure. Alternatively, as previously suggested, PDE inhibitors may induce relaxation of both the urethral smooth muscle and the striated external urethral sphincter, thereby potentially reducing urethral pressure.

Urethral pressure reflectometry (UPR) has emerged as a sensitive and reliable technique for assessing physiological parameters of urethral function. Its improved reproducibility compared with conventional urethral pressure profilometry allows for a more accurate investigation of pharmacodynamic changes in urethral pressure with fewer subjects [11].

To further elucidate the effect of PDE5 inhibitors on the female urethra, we used UPR to evaluate the effect of tadalafil, a PDE5 inhibitor, on opening urethral pressure in the resting state of the pelvic floor and during voluntary squeeze in healthy women. Additionally, we examined the effect of tadalafil on non-invasive urinary flow rates.

## Materials and Methods

After receiving approvals from the regional ethics committee (H-2100186) and the Danish Medicines Agency (EudraCT number 2020–005839-76), this trial, registered at www.ClinicalTrials.gov (ID NCT05095077), was conducted at the Zelo Phase 1 Unit, Copenhagen University Hospital Bispebjerg and Frederiksberg. The trial adhered to International Council for Harmonization Good Clinical Practice (GCP) guidelines and relevant legislation, and was overseen by the GCP Unit at Copenhagen University Hospital.

### Participants

Healthy female participants were recruited through advertisement on an online platform for trial participants (www.forsoegsperson.dk). Eligible participants were women between 18 and 55 years (both included) with a body weight of 50 kg or more and a body mass index (BMI) between 18.5 and 30 kg/m^2^. Other inclusion criteria included the use of safe contraceptive methods or sexual abstinence throughout the trial period. We excluded women with a history of urinary dysfunction, including incontinence, retention, or overactive bladder; clinically significant acute or chronic medical condition; systemic drug use within the 2 weeks before the first trial day (except for paracetamol [up to 4 g/day], hormonal contraception and hormone replacement therapy); smoking within the previous 3 months; pregnancy within the previous 6 months; and current breastfeeding. All participants provided written informed consent upon entering the trial.

### Trial Design, Randomization, and Masking

This was a randomized, double-blind, placebo-controlled, two-visit crossover trial with healthy female volunteers. Participants were randomly allocated (1:1) to receive either a blinded single dose of tadalafil (40 mg) or a visually identical placebo at their first visit and then all participants crossed over and received the opposite treatment at the second visit. The production and packaging of the blinded dosing kit and the randomization process were carried out by the hospital pharmacy. In this way, investigators, outcome assessors, data managers, and participants were blinded to the random sequence allocation throughout the trial period, data management, and statistical analysis. To avoid carry-over effects, the visits were separated by a washout period of a minimum of 6 days, corresponding to approximately eight half-lives of tadalafil, giving a mean terminal half-life of 17.5 h [12].

### Procedures and Equipment

At both clinic visits, participants underwent a human chorionic gonadotropin (hCG) test to rule out pregnancy before ingesting the study drug (40 mg of tadalafil or matching placebo). After 2 h of rest in the clinic, aligned with the median time to peak plasma concentration of tadalafil (t_max_) [12], the urethral pressure was assessed using UPR. The UPR technique involves continuous measurements of pressure and cross-sectional areas using a thin, flexible polyurethane bag. The participants were evaluated as previously described [13, 14]. With participants in the lithotomy position, the bladder was emptied by sterile urethral catheterization, followed by the introduction of 150 ml isotonic saline at 37°C. Ten urethral pressure measurements were performed while the participant was resting and five measurements during voluntary squeezes. Anal pressure was assessed using anal acoustic reflectometry (AAR) after UPR (the results from AAR measurement will be published separately). Immediately following UPR and AAR measurements, another 150 ml saline was instilled in the bladder, resulting in a standardized volume of 300 ml saline in the bladder. Subsequently, urinary flow rates and voided volume were recorded in undisturbed settings using the wireless uroflowmeter Flowmaster (Laborie, Portsmouth, NH, USA). Adverse events (AEs) were documented during clinic visits and follow-up telephone visits conducted at least 6 days after the last trial day.

### Outcomes

The primary endpoint was mean opening urethral pressure (OUP) during the resting condition of the pelvic floor for tadalafil versus placebo, whereas the secondary endpoint was mean OUP during the squeezing condition of the pelvic floor. Prespecified exploratory endpoints included peak urine flow rate (Q_max_), average urine flow rate (Q_ave_), and voided urine volume.

### Data Management, Statistical Analysis, and Sample Size Calculation

Study data were collected and managed using REDCap (Research Electronic Data Capture) hosted at the Capital Region of Denmark. REDCap is a secure, web-based software platform designed to support data capture for research studies [15, 16].

The sample size was calculated based on previous single-dose pharmacodynamic UPR trials [14, 17]. Assuming that the mean within-subject standard deviation (SD) was 9.7 cmH_2_O and using a two-tailed alpha of 0.05, a total of 24 participants would provide 99% power to detect a clinically relevant difference of 10 cmH_2_O in OUP.

The basic characteristics of the participants and AEs were evaluated descriptively. The differences in OUP (tadalafil versus placebo) and voiding parameters were analyzed using analysis of covariance (ANCOVA) models with participant ID, treatment, and period fitted as fixed effects. From these tests, least squares mean estimates between treatments, 95% confidence intervals (CIs), and *p* values are provided. All data management and statistical analyses were performed while blinded to the sequence allocation. We used SAS software, version 7.15 of the SAS system for Windows (SAS Institute, Cary, NC, USA), and GraphPad Prism version 9.4.1 for Windows (GraphPad Software, San Diego, CA, USA) for graphical plots.

## Results

### Participants

Twenty-four healthy female volunteers signed informed consent, were screened, and found to be eligible for inclusion in the trial. All 24 participants completed the protocol. The first participant was randomized on 8 August 2021, and the final date of observation for the last participant was 10 January 2022. The clinical characteristics of the 24 participants at baseline are shown in Table 1.

**Table 1:** Baseline clinical characteristics by sequence and by total

	Tadalafil to placebo (*n* = 12), *median (range)*	Placebo to tadalafil (*n* = 12), *median (range)*	Total (*N* = 24), *median (range)*
Age, years	25.0 (22.0–43.0)	24.0 (20.0–43.0)	24.5 (20.0–43.0)
Weight, kg	65.0 (55.0–76.0)	60.0 (53.0–75.0)	63.5 (53.0–76.0)
Body mass index, kg/m^2^	23.4 (19.0–26.9)	21.1 (19.0–26.6)	22.7 (19.0–26.9)
Washout, days	14 (7–26)	10 (7–77)	12 (7–77)

### Outcomes

**Fig. 1:**
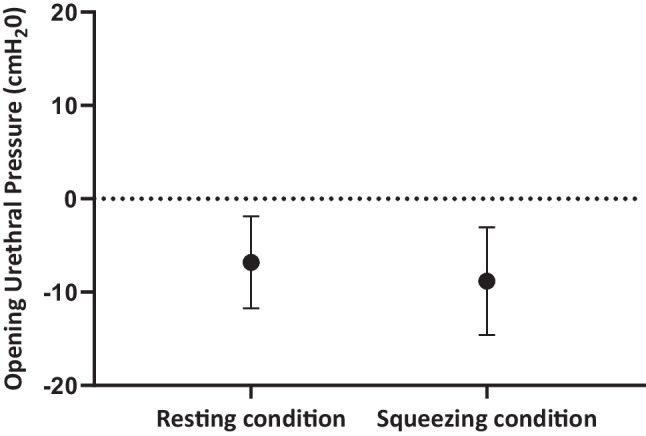
Effect estimates (95% confidence interval) of tadalafil versus placebo on opening urethral pressure during resting and squeezing condition of the pelvic floor

As shown in Fig. 1, a single 40-mg dose of tadalafil reduced mean resting OUP by 6.8 cmH_2_0 (-6.8, 95% confidence interval [CI] -11.8 to -1.9; *p* = 0.009) and mean squeezing OUP by 8.8 cmH_2_0 (-8.8, 95% CI -14.6 to -3.1; *p* = 0.005), compared with placebo.

**Fig. 2:**
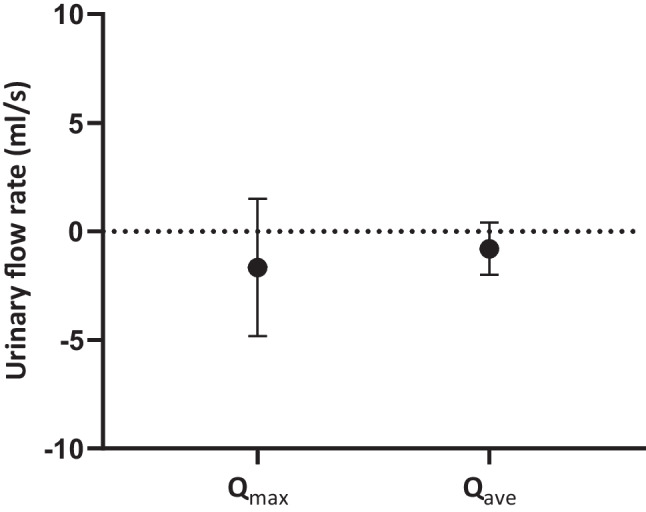
Effect estimates (95% confidence interval) of tadalafil versus placebo on peak urinary flow rate (Q_max_) and average urinary flow rate (Q_ave_)

No statistically significant differences in voiding parameters were found when comparing tadalafil with placebo (Q_max_ -1.7 ml/s [95% CI -4.8 to 1.5, *p* = 0.3]; Q_ave_ -0.8 ml/s [95% CI -2.0 to 0.4, *p* = 0.2]; voided volume −11 ml [95% CI -41 to 18.0, *p* = 0.4]; Fig. 2). Four participants voided less than 300 ml (range 261–297 ml) at one visit each (two following placebo administration), whereas the median voided volume was 341 ml (SD 58 ml).

### Harms

Out of a total of 41 reported AEs, 36 were reported on the tadalafil day and five on the placebo day. All AEs were documented and assessed by a blinded investigator. The most frequently reported AE was headache, with 15 cases associated with tadalafil and 3 with placebo. Most AEs were mild in severity, and no serious adverse events occurred. All reported AEs are listed in Table 2.

**Table 2:** Adverse events during treatment periods

	Tadalafil (*N* = 24), *n* (%)	Placebo (*N* = 24), *n* (%)
Headache	15 (63)	3 (13)
Migraine	1 (4)	–
Dizziness	2 (8)	1 (4)
Nausea	1 (4)	–
Fatigue	2 (8)	1 (4)
Nasal congestion	2 (8)	–
Flushing	8 (33)	–
Back pain	1 (4)	–
Photophobia	1 (4)	–
Eye swelling	1 (4)	–
Feeling of ear congestion	1 (4)	–
Genital swelling	1 (4)	–
Total	36	5

## Discussion

Among 24 healthy female participants, this trial found that a single oral dose of 40 mg of tadalafil caused a reduction in OUP compared with placebo. This reduction in urethral pressure, which was observed during both resting and squeezing conditions of the pelvic floor, suggests that tadalafil induces some relaxation of the female urethral musculature. These results support the findings from preclinical studies linking NO, cGMP, and PDE5 immunoreactivity in smooth and striated muscle cells from female urethral tissue to urethral smooth muscle relaxant actions of PDE5 inhibitors [5, 6]. Recently, the findings of PDE5 expression in the female urethra were confirmed and further expanded by Rahardjo et al. [18]. They found that all PDE isoenzymes examined in their Western blot experiments (PDE 1, −2, −4, −5, −10, and −11) were expressed in female urethral tissue specimens. As tadalafil has demonstrated an inhibitory effect of both PDE5 and, to some extent, of PDE11 [19], the combined inhibition of these enzymes may mediate relaxation of the female urethra. Further, it remains unclear whether the decrease in urethral pressure is a result of tadalafil-induced PDE inhibition affecting the striated external sphincter, solely due to the relaxation of smooth muscle (potentially including both urethral and vascular smooth muscle), or a combination of these factors. However, the expression of neuronal nitric oxide synthase in the external urethral sphincter suggests that the NO/cGMP pathways might play a role in the regulation of the striated urethral sphincter tonus [6].

We did not find any increases in urinary flow rates as a response to tadalafil, an expected consequence of urethral pressure reduction. There are several potential explanations for this inconsistency. Differences in urinary flow rates were explorative outcomes and, therefore, power calculations were not performed. Furthermore, the trial involved healthy, young women presumably entering the trial with an unrestricted urinary flow. This implies that any potential increase in urinary flow associated with a urethral pressure reduction may not be readily anticipated in this group of healthy volunteers. Finally, tadalafil may also induce relaxation of the detrusor smooth muscle. This relaxation could counterbalance the decreased urethral resistance caused by the reduction in urethral pressure [20].

With the caveat that a single-dose trial in healthy individuals should be interpreted with caution, the reduction in urethral pressure induced by tadalafil may have a clinical impact. For women with urinary retention the treatment goal is to ensure adequate bladder emptying [21]. As previously noted, the concept of augmenting the relaxation of the urethral sphincter through PDE5 inhibition to obtain improved bladder emptying is not novel. In the clinical crossover trial by Datta et al., which examined the effect of 50 mg of sildenafil twice daily for a duration of 4 weeks to women with Fowler’s syndrome, the study found no significant differences in urinary flow rates or symptom scores when compared with placebo [9]. However, it is important to note that the design of this trial differs from the current trial in several critical aspects. First, we used tadalafil, which may exert other pharmacodynamic effects on the female urethra owing to the higher activity against PDE11 compared with sildenafil [19]. As previously noted, PDE11 appears to be highly expressed in the female urethral tissue, supporting the notion that tadalafil could exert urethral sphincter relaxation via PDE11 inhibition. Second, because the trials involved different groups of participants, it is uncertain whether the decrease in urethral pressure caused by tadalafil in healthy women would have the same effect in individuals with urinary retention. Finally, the primary outcomes were different, precluding meaningful direct comparisons. Overall, tadalafil might have a different effect on the female urethra than sildenafil, and the potential clinical impact for women with urinary retention or related voiding dysfunctions is intriguing. Long-term clinical trials in these populations would be required to further evaluate the clinical potential. However, the magnitude of urethral pressure reduction obtained in the current trial was relatively small, suggesting that the efficacy may be limited.

Although the majority of AEs that emerged in this study were anticipated side effects to tadalafil, transient and mild in severity, the frequency of these tadalafil-related AEs was higher than that in longer-term studies involving male participants with erectile dysfunction [22] and premenopausal women with sexual dysfunction [23]. This increased frequency might be attributed to the study design, which involved a single high dose of the drug. In contrast, the referenced studies employed lower doses over an extended period, where the AE frequency generally appeared to decrease [22].

In addition to the restricted generalizability of the study results owing to the healthy study population, other limitations need to be considered. First, the urethral pressure measurements were specifically conducted during the storage phase. Consequently, we are unable to directly infer the impact of tadalafil on the female urethra during the voiding phase. Furthermore, the single-dose—and single-outcome measurement design—provided only a snapshot of the pharmacodynamic profile of tadalafil. If the maximal plasma concentration in some of the participants was reached substantially earlier or later than the anticipated 2 h according to the summary of product characteristics [12], the urethral pressure recordings may not have captured the maximum effect of tadalafil. Nevertheless, we do not expect that the direction of the effect would change, only the magnitude of the effect. Indeed, the trial design is based on our experience from previous single-dose UPR studies with healthy participants, demonstrating that the drugs under investigation exhibited their maximum effect on urethral pressure very close to the estimated t_max_ [14, 24]. Finally, we did not include sonographic evaluation of post-void residuals as part of our assessment of voiding function. Instead, we standardized the bladder volume by emptying the bladder, followed by the instillation of 300 ml (2 × 150 ml) of saline. The immediate recording of voided volume allowed us to estimate any potential post-void residuals. Although the participants typically voided more than the instilled volume, with only four exceptions (ranging from 261 to 297 ml), we cannot completely rule out minor post-void residuals due to the continuous diuresis occurring during the time lapse between bladder drainage and voiding. Nevertheless, given our crossover study design and clear instructions to the participants to maintain consistent fluid intake on both clinic visits, we anticipate that any such residuals would not significantly affect our results. In fact, our findings indicated no statistically significant differences in voided volume between the clinic visits (-11 ml [CI -41 to 18.0, *p* = 0.4]).

## Conclusions

To our knowledge, this is the first randomized, placebo-controlled trial assessing the effect of tadalafil on urethral pressure in women. A single-dose (40 mg) tadalafil resulted in a placebo-corrected reduction in the opening urethral pressure in healthy women both during the resting state and during voluntary contraction of the pelvic floor. This finding suggests that PDE5 (and to some extent PDE11) inhibition might induce relaxation of the female urethra. The clinical implication of this reduction remains to be elucidated.

## Data Availability

The data that support the findings of this study are not openly available due to reasons of sensitivity and are available from the corresponding author upon reasonable request. Data are located in controlled access data storage at Copenhagen University Hospital - Bispebjerg and Frederiksberg.
